# Pathogenesis and therapeutic strategies for neuropsychiatric lupus centered on innate immune activation

**DOI:** 10.1007/s13577-026-01381-5

**Published:** 2026-04-16

**Authors:** Wataru Nagata, Toshiaki Ishizuka

**Affiliations:** 1 Department of Pharmacology, National Defense Medical College, 3-2, Namiki, Tokorozawa, Saitama 359-0042 Japan; 2 Division of Environmental Medicine, National Defense Medical College, NDMC Research Institute, 3-2, Namiki, Tokorozawa, Saitama 359-0042 Japan

**Keywords:** Systemic lupus erythematosus, Neuropsychiatry, Immunity, Innate, Interferon type I, Blood–brain barrier

## Abstract

Neuropsychiatric systemic lupus erythematosus (NPSLE) is a serious central nervous system complication of systemic lupus erythematosus (SLE) that markedly reduces patient quality of life. Despite its clinical importance, the underlying mechanisms remain incompletely defined, and effective treatments are limited. In this review, we synthesize preclinical and clinical evidence that aberrant activation of innate immunity by self-nucleic acids and consequent overproduction of Type I interferons (IFN-I) constitute a central pathogenic axis in NPSLE. IFN-I and other inflammatory mediators promote disruption of the blood–brain barrier (BBB), enabling entry of autoantibodies, cytokines, and immune cells into the brain. These factors, together with damage-associated molecular patterns, activate microglia and astrocytes, driving sustained neuroinflammation that provokes synaptic loss, neurotransmitter dysregulation, excitotoxic neuronal injury, impaired neurogenesis, and mitochondrial dysfunction—mechanisms that underlie cognitive impairment, mood disorders, and other neuropsychiatric manifestations. We review therapeutic strategies targeting each step of this cascade, including blockade of IFN-I signaling (e.g., anifrolumab), inhibition of endosomal nucleic acid sensing (TLR antagonists), cytokine and JAK inhibition, modulation of microglial function (CSF1R inhibitors), and approaches to protect or restore BBB integrity (e.g., statins). Finally, we discuss biomarker-guided patient stratification and trial designs necessary to address NPSLE heterogeneity and accelerate the development of personalized therapies. By elucidating the cellular responses of the neurovascular unit to innate immune insults, this review provides a molecular framework for developing targeted therapies for NPSLE.

## Introduction

The central nervous system (CNS) was historically considered immune-privileged, but this notion has been revised in recent years [1]. Remarkable advances in psychoneuroimmunology now indicate that neuroinflammation driven by the brain’s resident immune cells, principally microglia, can constitute a common pathogenic substrate for diverse neuropsychiatric disorders, including depression, schizophrenia, and dementia [2, 3]. This paradigm shift is important for understanding how systemic autoimmune diseases may directly impact brain function.

Systemic lupus erythematosus (SLE) is a systemic autoimmune disease characterized by immune responses to self-nucleic acids, with chronic activation of the innate immune system, in particular the Type I interferon (IFN-I) pathway [4, 5]. Patients with SLE frequently develop diverse neuropsychiatric manifestations, collectively referred to as neuropsychiatric SLE (NPSLE) [6].　The American College of Rheumatology (ACR) classifies NPSLE into 19 syndromes that range from severe neurological events (for example, seizures and stroke) to psychiatric presentations, including cognitive dysfunction, depression, and anxiety [7]. Cognitive decline and persistent mood disorders markedly reduce quality of life (QOL) and frequently lead to withdrawal from work and social activities [7]. Severe neurological complications, such as seizures and stroke, are associated with permanent disability and increased mortality [8].

Historically, high-dose corticosteroids and broad-spectrum immunosuppressants (e.g., cyclophosphamide) have been the mainstays of NPSLE treatment [9, 10]. However, many immunosuppressive agents have limited penetration across the blood–brain barrier (BBB), restricting their direct effects on intracerebral inflammation and neuronal injury [11]. Moreover, broad immunosuppression increases the risk of serious adverse events—including infections, avascular necrosis, and steroid-induced psychiatric effects—which can further worsen QOL [12]. Thus, conventional therapies often do not provide sufficient symptom control or QOL improvement.

**Fig. 1:**
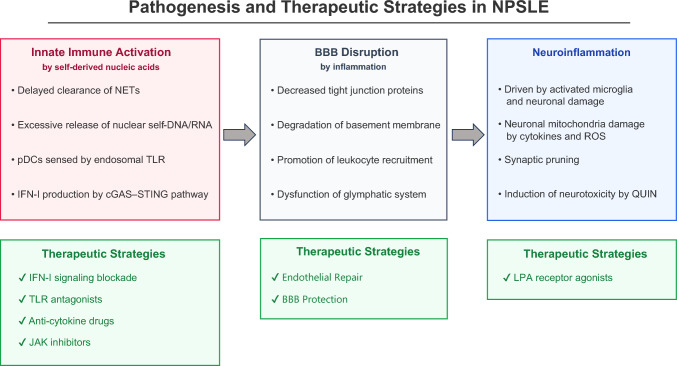
Schematic representation of the pathogenesis and emerging therapeutic targets in neuropsychiatric systemic lupus erythematosus (NPSLE). Aberrant activation of innate immunity by self-nucleic acids and the subsequent overproduction of Type I interferons (IFN-I) constitute a central pathogenic axis. Chronic IFN-I signaling and other inflammatory mediators promote the disruption of the blood–brain barrier (BBB), facilitating the entry of autoantibodies and peripheral immune cells into the brain parenchyma. Within the central nervous system, these factors, along with damage-associated molecular patterns (DAMPs), activate glial cells (microglia, astrocytes, and oligodendrocytes), leading to sustained neuroinflammation, synaptic loss, and neuronal dysfunction. Potential therapeutic interventions, including IFNAR inhibitors, JAK inhibitors, and modulators of LPA signaling, aim to interrupt these pathogenic cascades at various stages

In this review, we first describe how aberrant activation of innate immunity in SLE promotes IFN-I-mediated disruption of the BBB, entry of peripheral inflammatory mediators into the CNS, activation of glial cells, and subsequent neuronal injury—processes that underlie cognitive impairment, mood disorders, and other neuropsychiatric manifestations. We then summarize emerging therapeutic approaches that target discrete steps of this pathogenic cascade and discuss strategies for biomarker-guided patient stratification and trial design. The integrated pathogenic axis and therapeutic targets discussed in this review are schematically illustrated in Fig. 1.

## The impact of innate immune activation on NPSLE pathogenesis

### Aberrant activation of innate immunity via nucleic acid sensors and consequent upregulation of Type I interferon production

Abnormal stimulation of the immune system by self-derived nucleic acids is central to SLE pathogenesis. In patients with SLE, clearance of apoptotic debris and neutrophil extracellular traps (NETs), which is rapid in healthy individuals, is delayed [13, 14]. This delay leads to excess circulating immune complexes and extracellular vesicles containing self-DNA and RNA [15]. These self-nucleic acids are internalized by innate immune cells such as plasmacytoid dendritic cells (pDCs) and are sensed by endosomal Toll-like receptors (TLR7 and TLR9) [16, 17]. In the cytosol, the cGAS–STING pathway functions as a DNA-sensing axis: cGAS detects aberrant cytosolic DNA and synthesizes the second messenger cyclic GMP–AMP (cGAMP), which activates STING and triggers IFN-I production via TBK1–IRF3 signaling　[17]. This pathway senses self-DNA derived from the nucleus or mitochondria as well as pathogen DNA; its aberrant activation is a key driver of autoimmune diseases such as SLE. Signals from these nucleic acid sensors activate IRF7 in pDCs, resulting in sustained high-level production of IFN-I, particularly IFN-*α* [18, 19]. The resulting upregulation of interferon-stimulated genes (the “IFN signature”) is a prominent feature in the peripheral blood of many SLE patients and correlates with disease activity and organ involvement [20]. Circulating IFN-*α* can act on brain vascular endothelial cells to increase BBB vulnerability; some studies suggest that IFN-*α* may cross the BBB and influence intracerebral immune responses [21, 22].

### Functional disruption of the BBB by inflammation

The healthy blood–brain barrier (BBB) is formed by the neurovascular unit (NVU), comprising brain capillary endothelial cells, the underlying basement membrane, pericytes, and astrocytic end-feet [23, 24]. The NVU is a highly selective barrier that protects the brain microenvironment by restricting entry of circulating immune cells, inflammatory mediators (for example, cytokines), and other potentially harmful substances [25]. It also contributes to nutrient transport and waste clearance. In NPSLE, peripherally derived cytokines, such as IFN-*α*, TNF-*α*, and IL-1*β*, act on multiple NVU components [26]. These cytokines decrease expression of junctional proteins—notably the tight-junction protein claudin-5 and the adherens-junction protein VE-cadherin—thereby creating gaps in the barrier [27, 28]. Concurrently, endothelial cells upregulate adhesion molecules, such as VCAM-1 and ICAM-1, promoting recruitment and transmigration of activated leukocytes [26]. Inflammatory signaling also induces matrix metalloproteinases (notably MMP-9), which degrade the basement membrane and promote structural disruption of the BBB [29, 30].

Once the BBB is compromised, plasma components, including albumin, autoantibodies, and complement, can leak into the brain parenchyma. Dysfunction of the glymphatic system—the brain’s waste-clearance pathway that depends on aquaporin-4 (AQP4) channels expressed on astrocytic end-feet—may further impair removal of inflammatory by-products, exacerbating neuroinflammation [31–35]. Reports of elevated IFN-*α* activity and increased IL-6 in serum and cerebrospinal fluid (CSF) of NPSLE patients [36], together with findings in the MRL/lpr mouse model showing elevated IL-6 and cognitive deficits that are ameliorated by IL-6 knockout [36, 37], suggest that intracerebral accumulation of inflammatory by-products and neurotoxic metabolites may represent an important pathogenic mechanism in NPSLE. Thus, BBB disruption and glymphatic dysfunction can act synergistically to compromise brain homeostasis and amplify neuroinflammation.

### Neuroinflammation driven by activated glial cells

Inflammatory mediators that enter the brain, together with damage-associated molecular patterns (DAMPs) such as extracellular ATP and HMGB1 released from dying cells, potently activate resident immune cells—microglia and astrocytes, and oligodendrocytes. While microglia and astrocytes have been the primary focus in NPSLE, emerging evidence suggests that oligodendrocytes, which are responsible for myelin formation, also suffer from innate immune insults, contributing to white matter distress and cognitive symptoms [38–41]. DAMPs are endogenous molecules released upon cellular injury that trigger innate immune responses via pattern recognition receptors (PRRs) [42, 43]. For example, extracellular ATP signals via purinergic receptors to promote inflammation, and extracellular HMGB1 induces cytokine release [44]. In SLE, delayed clearance of apoptotic cells can sustain extracellular DAMP levels [12], and evidence indicates that DAMPs directly induce microglial activation. Extracellular HMGB1 can bind microglial receptors (e.g., Mac1), activate NF-κB signaling, and induce production of neurotoxic inflammatory mediators, potentially creating a self-amplifying cycle of chronic neuroinflammation and progressive neurodegeneration [45, 46]. HMGB1 has also been reported to facilitate complement protein C1q binding to NMDA receptors and thereby enhance synapse elimination, with consequent spatial memory deficits in experimental settings [47]. Moreover, microglia-derived cytokines can shift astrocytic transcriptional programs toward a reactive, potentially neurotoxic phenotype, further impairing neuronal function [48]. Through such DAMP-mediated interactions, microglia and astrocytes form an amplifying immune network that contributes to NPSLE progression.

#### Microglia

In response to stimuli such as IFN-I or LPS, microglia adopt a pro-inflammatory phenotype and produce cytokines (IL-1*β*, IL-6, and TNF-*α*) as well as reactive oxygen and nitrogen species (ROS and NO) [49, 50]. Activation of the NLRP3 inflammasome matures IL-1*β* and IL-18 via caspase-1, further amplifying inflammation [51]. In this neuroinflammatory process, it is crucial to distinguish between resident microglia, which originate from yolk sac progenitors, and bone marrow-derived macrophages that infiltrate the CNS following BBB disruption, as their functional contributions to tissue injury and repair may differ [2, 52].

#### Astrocytes

Once considered mainly supportive cells, astrocytes play active roles in inflammation [53]. Reactive astrocytes release chemokines (e.g., CCL2 and CXCL10) that promote peripheral immune cell recruitment, sustaining and expanding the inflammatory milieu [54]. Although astrocyte-derived glial scars can limit propagation of damage, they may also inhibit neural regeneration [55].

#### Oligodendrocytes

Although microglia and astrocytes have been the primary focus of neuroinflammation research in NPSLE, oligodendrocytes—the myelin-forming cells of the CNS—are increasingly recognized as active participants in innate immune responses. Oligodendrocytes and their precursors express pattern recognition receptors including Toll-like receptors and are vulnerable to inflammatory cytokines, such as TNF-α and IFN-γ, which can impair differentiation, induce oxidative stress, and trigger apoptosis [40]. Loss of oligodendrocyte integrity disrupts myelin sheaths, impairing axonal conduction and contributing to the white matter abnormalities and cognitive deficits observed in NPSLE patients. Emerging evidence thus suggests that oligodendrocyte damage is not merely a bystander effect of neuroinflammation, but an active component of NPSLE pathology that warrants further investigation.

Activation of microglia, astrocytes, and oligodendrocytes generates a feed-forward loop of neuronal injury and additional DAMP release, propagating chronic neuroinflammation that underlies CNS pathology in NPSLE.

### Molecular mechanisms of neuronal dysfunction

Sustained glia-driven neuroinflammation impairs neurons and causes neuropsychiatric symptoms via multiple mechanisms.

#### Synaptic impairment and cognitive decline

Activated microglia can excessively prune synapses, a process linked to complement proteins C1q and C3 acting as opsonins that target synapses for microglial phagocytosis [56–59]. In animal models of NPSLE, IFN-I has been shown to enhance complement-mediated synapse elimination, decreasing hippocampal synaptic density and impairing cognition [59]. Human data directly demonstrating synapse loss in NPSLE are limited, but PET imaging (SV2A) in related neuroinflammatory diseases such as multiple sclerosis reveals cortical and hippocampal synaptic loss, suggesting a plausible parallel [60]. This finding suggests that similar synaptic loss may occur in NPSLE patients. The loss of synapses in brain regions critical for learning, memory, and executive function, such as the hippocampus and prefrontal cortex, is considered a direct cause of these cognitive impairments. The convergence of multiple pathways leading to cognitive dysfunction reflects the complex pathology of NPSLE.

#### Neurotransmitter dysregulation and mood disorders

Inflammatory cytokines can inhibit tryptophan hydroxylase and induce indoleamine 2,3-dioxygenase (IDO1), diverting tryptophan into the kynurenine pathway and producing metabolites, such as kynurenic acid (KYNA) and quinolinic acid (QUIN) [3, 61, 62]. QUIN, produced primarily by activated microglia and macrophages, is an NMDA receptor agonist that may induce neuronal hyperexcitability and neurotoxicity. Elevated QUIN has been associated with depressive symptoms in patients undergoing IFN-*α* therapy, and preclinical NPSLE models implicate this pathway in depressive-like and anxiety-like behaviors [63–66].

#### Excitotoxicity and cell death

Astrocytic dysfunction—for example, impaired expression or activity of the glutamate transporter GLT-1—can reduce glutamate clearance, leading to excessive NMDA receptor activation, intracellular Ca2 + overload, mitochondrial dysfunction, and activation of proteases such as calpains, culminating in neuronal death [67]. Anti-NMDA receptor antibodies that penetrate the CNS may bind receptors, alter receptor trafficking, or promote receptor overactivation, thereby exacerbating excitotoxic injury [68–70].

#### Suppression of neurogenesis and mood disorders

The dentate gyrus of the hippocampus is a site of adult neurogenesis that contributes to learning, stress resilience, and mood regulation. Neuroinflammation potently suppresses proliferation and differentiation of neural progenitor cells, thereby inhibiting hippocampal neurogenesis; this suppression is implicated in depressive-like behaviors and increased vulnerability to stress [71, 72]. In the MRL/lpr mouse model of NPSLE, heightened neuroinflammation is associated with reduced hippocampal neurogenesis, depressive-like behaviors, and cognitive deficits, effects that are at least partly reversible in experimental manipulations [73]. In humans, MRI studies reporting hippocampal atrophy in some NPSLE patients provide indirect support for impaired neurogenesis or neuronal loss, although direct evidence linking suppressed neurogenesis to mood disorders in patients remains limited [74]. Thus, suppression of neurogenesis is a plausible mechanism contributing to mood changes in NPSLE, but further translational and clinical studies are needed to clarify causality and therapeutic implications.

#### Mitochondrial dysfunction and oxidative stress

Activated microglia produce reactive oxygen species (ROS) and reactive nitrogen species such as nitric oxide (NO), which can directly damage neuronal mitochondria. These insults lead to mitochondrial DNA damage, impairment of electron transport chain complexes, loss of membrane potential, and increased mitochondrial permeability, culminating in reduced ATP production and energetic failure [75]. Energy deficits sensitize neurons to excitotoxic and apoptotic pathways, amplifying inflammation-driven neuronal injury and creating a feed-forward loop between mitochondrial dysfunction and neuroinflammation [50]. Notably, mitochondrial dysfunction and increased oxidative stress have also been documented in immune cells from patients with SLE, suggesting systemic alterations in redox and mitochondrial biology that may influence CNS vulnerability [75]. Targeting oxidative stress and supporting mitochondrial function are, therefore, plausible adjunctive strategies to mitigate neuronal injury in NPSLE.

## Therapeutic strategies for NPSLE based on pathogenic mechanisms

Managing NPSLE remains a significant clinical challenge, often requiring a multidisciplinary approach that combines immunosuppressive therapy with symptomatic management. Based on the evolving understanding of innate immune-mediated pathogenesis, several novel therapeutic strategies have been proposed. These current and emerging therapeutic strategies for NPSLE, including their molecular targets, mechanisms of action, and clinical development status, are summarized in Table 1.

**Table 1:** Summary of therapeutic strategies and agents for NPSLE

Category	Therapeutic agent	Target/mechanism	Development status in NPSLE/SLE
Biologics	Anifrolumab	Type I IFN receptor (IFNAR)	Approved for SLE; clinical trials for NPSLE ongoing
	Belimumab	B-cell activating factor (BAFF)	Approved for SLE; potential CNS benefit
Small molecules	JAK inhibitors	JAK1/2/3 signaling (IFN signaling)	Case reports and pilot studies show efficacy
	Statins	Mevalonate pathway/anti-inflammatory	Neuroprotective in preclinical models
Lipid modulators	2-Carba cyclic phosphatidic acid (2ccPA)/modulators of LPA signaling	LPA receptor signaling/microglial switch	Preclinical evidence for neuroprotection

### Suppressing innate immune activation

Targeting upstream drivers of the pathogenic cascade aims to prevent downstream BBB disruption and CNS inflammation.

#### IFN-I signaling blockade

Because Type I interferons are central to SLE immunopathology, blocking IFN-I signaling is a rational strategy for NPSLE. Anifrolumab, a monoclonal antibody against the type I IFN receptor, has shown efficacy in reducing systemic disease activity in phase III trials [76]. Its potential benefit for NPSLE is plausible and under investigation. When discussing IFN-I blockade, it is important to distinguish systemic effects from direct CNS effects and to consider safety signals reported in trials (for example, infection risk).

#### Inhibition of endosomal nucleic acid sensing (TLR antagonists)

Agents that inhibit TLR7/8/9 signaling can reduce pDC activation and IFN-I production. Hydroxychloroquine exerts partial inhibition of endosomal TLR signaling and is a standard therapy in SLE; more selective TLR antagonists are in development and may offer greater specificity [77].

### Attenuating neuroinflammation

Interventions that dampen CNS inflammation or modulate glial responses aim to limit neuronal injury once peripheral immune activation or BBB disruption has occurred.

#### Cytokine inhibition

Targeting key inflammatory cytokines implicated in CNS pathology is supported by preclinical data and case reports. IL-6 receptor antagonists (e.g., tocilizumab and satralizumab) and IL-1 pathway blockers (e.g., anakinra) have been used for refractory neuroinflammatory presentations [78–80]. Evidence in NPSLE remains limited and largely observational, so controlled studies are needed.

#### JAK inhibition

Janus kinase inhibitors (for example, baricitinib and upadacitinib) broadly inhibit signaling downstream of multiple cytokines, including IFN-I. Clinical trials demonstrate efficacy in systemic SLE [81, 82]. Oral small molecules may achieve better CNS penetration than large biologics, making JAK inhibitors attractive for NPSLE; however, potential class-specific adverse effects (e.g., thromboembolic risk and infection) warrant careful evaluation [83].

#### Modulation of microglial function

Strategies to selectively reduce proinflammatory microglia or shift them toward neuroprotective phenotypes (for instance, CSF1R inhibitors or other modulators) have shown disease-modifying effects in neurodegeneration models [84, 85]. Preclinical NPSLE data are encouraging, but translation to humans requires demonstration of safety, target engagement, and functional benefit.

### Protecting and repairing the BBB

Preserving BBB integrity can prevent peripheral inflammatory mediators and pathogenic antibodies from accessing the CNS.

#### Vascular-protective/anti-inflammatory agents

Approaches to prevent BBB breakdown and restore its function should be pursued concurrently. Basic research suggests that the pleiotropic vasoprotective and anti-inflammatory effects of statins may contribute to the stabilization of BBB function [86, 87]. Specifically, simvastatin, a type of statin, has been reported to increase the expression of claudin-5, a tight junction protein in vascular endothelial cells [86]. Claudin-5 is a key component for the strict regulation of permeability at the BBB, and its upregulation strengthens the barrier function, thereby inhibiting the entry of peripheral inflammatory factors and harmful substances into the brain [88]. This effect is suggested to be mediated via the translocation of VE-cadherin to the membrane [89, 90], shedding light on one of the molecular mechanisms by which statins stabilize the BBB.

### Lysophosphatidic acid (LPA) receptors: a complex target with duality

Recently, it has been reported that receptors for lysophosphatidic acid (LPA), a type of lysophospholipid, particularly the LPA1 receptor, are widely expressed in the brain and that their activation exhibits antidepressant-like effects, making LPA1 agonists potential candidates for novel antidepressants [91]. Kariuki et al. conducted a genome-wide association study (GWAS) stratifying SLE patients by serological features and reported that a polymorphism within the *LPAR1* gene, which codes for the LPA1 receptor, is significantly associated with the production of anti-Sm antibodies and elevated blood IFN-α levels, both deeply involved in SLE pathogenesis [92]. This finding suggests that aberrant intracellular signaling through the LPA1 receptor may be involved in SLE pathogenesis via the expression of the IFN-I signature. We have shown that administration of LPA to the NPSLE model MRL/lpr mouse not only improves glomerulonephritis [93] but also exerts a neuroinflammation-suppressing effect [94]. On the other hand, there are also reports that LPA activates cell migration and inflammation [95]. Therefore, a more sophisticated strategy is required, such as understanding this complex duality and targeting autotaxin, the enzyme that produces LPA [96]. By inhibiting autotaxin, it may be possible to reduce the total amount of LPA, thereby suppressing its pro-inflammatory effects while maintaining the beneficial effects mediated by the LPA1 receptor, or to selectively modulate specific LPA receptors. In a related finding, 2-carba-cyclic phosphatidic acid (2ccPA) has been shown to promote the shift of microglia to an anti-inflammatory phenotype and improve depressive-like behavior in a mouse model of NPSLE [97]. This highlights the importance of modulating the balance of the LPA pathway rather than completely blocking it.

## Conclusion and future perspectives

The molecular mechanisms of NPSLE are becoming clearer, with evidence showing that aberrant innate immune activation and the IFN signature intrinsic to SLE induce BBB disruption and neuroinflammation, leading to neuropsychiatric symptoms via neuronal death and synaptic damage. This understanding is enabling new therapeutic approaches for NPSLE that target each stage of its pathogenesis.

A key future challenge is to overcome the clinical heterogeneity of NPSLE. In the previous reports, the diverse neuropsychiatric manifestations in SLE necessitate precise mapping of clinical phenotypes against serological and immunological markers [98]. It is essential to establish biomarkers (from blood, CSF, or imaging) to identify which pathogenic mechanisms are dominant in individual patients, which will allow for the personalization of the most promising treatments. Furthermore, designing clinical trials for NPSLE has been extremely difficult due to the diversity of symptoms and the challenges of objective evaluation. Future trials will require more sophisticated designs utilizing stratification biomarkers. For example, an approach is needed where patients are stratified based on biomarkers—such as applying IFN-I pathway inhibitors to the group with a high IFN signature, antibody removal therapies for those positive for anti-NMDA receptor antibodies, and IL-6 inhibitors for patients with high CSF IL-6 levels—to evaluate the efficacy of treatments tailored to each subgroup. This strategy is expected to maximize therapeutic effects and minimize side effects.

Future research directions include further elucidating how genetic susceptibility, environmental factors, and immune dysregulation interact in the onset of NPSLE; developing non-invasive, reliable biomarkers for early diagnosis and monitoring of CNS involvement; evaluating the long-term safety and efficacy of novel targeted therapies, especially their CNS permeability and impact on neural repair; and exploring the potential of combination therapies that act on multiple pathogenic pathways simultaneously. These advances are expected to improve the prognosis and QOL for patients with NPSLE.

## Data Availability

Data sharing is not applicable to this article as no new data were created or analyzed in this study.

## References

[1] Galea I, Bechmann I, Perry VH. What is immune privilege (not)? Trends Immunol. 2007;28(1):12–8. 10.1016/j.it.2006.11.004. 17129764 10.1016/j.it.2006.11.004

[2] Prinz M, Priller J. Microglia and brain macrophages in the molecular age: from origin to neuropsychiatric disease. Nat Rev Neurosci. 2014;15(5):300–12. 10.1038/nrn3722. 24713688 10.1038/nrn3722

[3] Dantzer R, O’Connor JC, Freund GG, Johnson RW, Kelley KW. From inflammation to sickness and depression: when the immune system subjugates the brain. Nat Rev Neurosci. 2008;9(1):46–56. 10.1038/nrn2297. 18073775 10.1038/nrn2297 PMC2919277

[4] Tsokos GC. Systemic lupus erythematosus. N Engl J Med. 2011;365(22):2110–21. 10.1056/NEJMra1100359. 22129255 10.1056/NEJMra1100359

[5] Rönnblom L, Eloranta ML, Alm GV. The type I interferon system in systemic lupus erythematosus. Arthritis Rheum. 2006;54(2):408–20. 10.1002/art.21571. 16447217 10.1002/art.21571

[6] Jeltsch-David H, Muller S. Neuropsychiatric systemic lupus erythematosus: pathogenesis and biomarkers. Nat Rev Neurol. 2014;10(10):579–96. 10.1038/nrneurol.2014.148. 25201240 10.1038/nrneurol.2014.148

[7] Unterman A, Nolte JE, Boaz M, Abady M, Shoenfeld Y, Zandman-Goddard G. Neuropsychiatric syndromes in systemic lupus erythematosus: a meta-analysis. Semin Arthritis Rheum. 2011;41(1):1–11. 10.1016/j.semarthrit.2010.08.001. 20965549 10.1016/j.semarthrit.2010.08.001

[8] Zirkzee EJ, Huizinga TW, Bollen EL, et al. Mortality in neuropsychiatric systemic lupus erythematosus (NPSLE). Lupus. 2014;23(1):31–8. 10.1177/0961203313512540. 24243776 10.1177/0961203313512540

[9] Bertsias GK, Ioannidis JP, Aringer M, et al. EULAR recommendations for the management of systemic lupus erythematosus with neuropsychiatric manifestations: report of a task force of the EULAR standing committee for clinical affairs. Ann Rheum Dis. 2010;69(12):2074–82. 10.1136/ard.2010.130476. 20724309 10.1136/ard.2010.130476

[10] Barile-Fabris L, Ariza-Andraca R, Olguín-Ortega L, et al. Controlled clinical trial of IV cyclophosphamide versus IV methylprednisolone in severe neurological manifestations in systemic lupus erythematosus. Ann Rheum Dis. 2005;64(4):620–5. 10.1136/ard.2004.025528. 15769918 10.1136/ard.2004.025528 PMC1755456

[11] Schwartz N, Stock AD, Putterman C. Neuropsychiatric lupus: new mechanistic insights and future treatment directions. Nat Rev Rheumatol. 2019;15(3):137–52. 10.1038/s41584-018-0156-8. 30659245 10.1038/s41584-018-0156-8 PMC8023338

[12] Kaplan MJ. Role of neutrophils in systemic autoimmune diseases. Arthritis Res Ther. 2013;15(5):219. 10.1186/ar4325. 24286137 10.1186/ar4325 PMC3978765

[13] Hakkim A, Fürnrohr BG, Amann K, et al. Impairment of neutrophil extracellular trap degradation is associated with lupus nephritis. Proc Natl Acad Sci U S A. 2010;107(21):9813–8. 10.1073/pnas.0909927107. 20439745 10.1073/pnas.0909927107 PMC2906830

[14] Gaipl US, Munoz LE, Grossmayer G, et al. Clearance deficiency and systemic lupus erythematosus (SLE). J Autoimmun. 2007;28(2–3):114–21. 10.1016/j.jaut.2007.02.005. 17368845 10.1016/j.jaut.2007.02.005

[15] Marshak-Rothstein A. Toll-like receptors in systemic autoimmune disease. Nat Rev Immunol. 2006;6(11):823–35. 10.1038/nri1957. 17063184 10.1038/nri1957 PMC7097510

[16] Christensen SR, Shupe J, Nickerson K, Kashgarian M, Flavell RA, Shlomchik MJ. Toll-like receptor 7 and TLR9 dictate autoantibody specificity and have opposing inflammatory and regulatory roles in a murine model of lupus. Immunity. 2006;25(3):417–28. 10.1016/j.immuni.2006.07.013. 16973389 10.1016/j.immuni.2006.07.013

[17] Ablasser A, Chen ZJ. cGAS in action: Expanding roles in immunity and inflammation. Science. 2019. 10.1126/science.aat8657. 30846571 10.1126/science.aat8657

[18] Siegal FP, Kadowaki N, Shodell M, et al. The nature of the principal type 1 interferon-producing cells in human blood. Science. 1999;284(5421):1835–7. 10.1126/science.284.5421.1835. 10364556 10.1126/science.284.5421.1835

[19] Baechler EC, Batliwalla FM, Karypis G, et al. Interferon-inducible gene expression signature in peripheral blood cells of patients with severe lupus. Proc Natl Acad Sci U S A. 2003;100(5):2610–5. 10.1073/pnas.0337679100. 12604793 10.1073/pnas.0337679100 PMC151388

[20] Crow MK. Type I interferon in the pathogenesis of lupus. J Immunol. 2014;192(12):5459–68. 10.4049/jimmunol.1002795. 24907379 10.4049/jimmunol.1002795 PMC4083591

[21] Campbell IL, Krucker T, Steffensen S, et al. Structural and functional neuropathology in transgenic mice with CNS expression of IFN-alpha. Brain Res. 1999;835(1):46–61. 10.1016/s0006-8993(99)01328-1. 10448195 10.1016/s0006-8993(99)01328-1

[22] Abbott NJ, Patabendige AA, Dolman DE, Yusof SR, Begley DJ. Structure and function of the blood–brain barrier. Neurobiol Dis. 2010;37(1):13–25. 10.1016/j.nbd.2009.07.030. 19664713 10.1016/j.nbd.2009.07.030

[23] Daneman R, Prat A. The blood–brain barrier. Cold Spring Harb Perspect Biol. 2015;7(1):a020412. 10.1101/cshperspect.a020412. 25561720 10.1101/cshperspect.a020412 PMC4292164

[24] Abbott NJ, Rönnbäck L, Hansson E. Astrocyte–endothelial interactions at the blood–brain barrier. Nat Rev Neurosci. 2006;7(1):41–53. 10.1038/nrn1824. 16371949 10.1038/nrn1824

[25] Iadecola C. The neurovascular unit coming of age: a journey through neurovascular coupling in health and disease. Neuron. 2017;96(1):17–42. 10.1016/j.neuron.2017.07.030. 28957666 10.1016/j.neuron.2017.07.030 PMC5657612

[26] Varatharaj A, Galea I. The blood–brain barrier in systemic inflammation. Brain Behav Immun. 2017;60:1–12. 10.1016/j.bbi.2016.03.010. 26995317 10.1016/j.bbi.2016.03.010

[27] Luissint AC, Artus C, Glacial F, Ganeshamoorthy K, Couraud PO. Tight junctions at the blood–brain barrier: physiological architecture and disease-associated dysregulation. Fluids Barriers CNS. 2012;9(1):23. 10.1186/2045-8118-9-23. 23140302 10.1186/2045-8118-9-23 PMC3542074

[28] Stamatovic SM, Keep RF, Andjelkovic AV. Brain endothelial cell–cell junctions: how to “open” the blood–brain barrier. Curr Neuropharmacol. 2008;6(3):179–92. 10.2174/157015908785777210. 19506719 10.2174/157015908785777210 PMC2687937

[29] Rosenberg GA. Matrix metalloproteinases and their multiple roles in neurodegenerative diseases. Lancet Neurol. 2009;8(2):205–16. 10.1016/s1474-4422(09)70016-x. 19161911 10.1016/S1474-4422(09)70016-X

[30] Harhaj NS, Antonetti DA. Regulation of tight junctions and loss of barrier function in pathophysiology. Int J Biochem Cell Biol. 2004;36(7):1206–37. 10.1016/j.biocel.2003.08.007. 15109567 10.1016/j.biocel.2003.08.007

[31] Plog BA, Nedergaard M. The glymphatic system in central nervous system health and disease: past, present, and future. Annu Rev Pathol. 2018;13:379–94. 10.1146/annurev-pathol-051217-111018. 29195051 10.1146/annurev-pathol-051217-111018 PMC5803388

[32] Iliff JJ, Wang M, Liao Y, et al. A paravascular pathway facilitates CSF flow through the brain parenchyma and the clearance of interstitial solutes, including amyloid β. Sci Transl Med. 2012. 10.1126/scitranslmed.3003748. 22896675 10.1126/scitranslmed.3003748 PMC3551275

[33] Louveau A, Smirnov I, Keyes TJ, et al. Structural and functional features of central nervous system lymphatic vessels. Nature. 2015;523(7560):337–41. 10.1038/nature14432. 26030524 10.1038/nature14432 PMC4506234

[34] Kress BT, Iliff JJ, Xia M, et al. Impairment of paravascular clearance pathways in the aging brain. Ann Neurol. 2014;76(6):845–61. 10.1002/ana.24271. 25204284 10.1002/ana.24271 PMC4245362

[35] Jessen NA, Munk AS, Lundgaard I, Nedergaard M. The glymphatic system: a beginner’s guide. Neurochem Res. 2015;40(12):2583–99. 10.1007/s11064-015-1581-6. 25947369 10.1007/s11064-015-1581-6 PMC4636982

[36] Reynolds J, Huang M, Li Y, et al. Constitutive knockout of interleukin-6 ameliorates memory deficits and entorhinal astrocytosis in the MRL/lpr mouse model of neuropsychiatric lupus. J Neuroinflamm. 2024;21(1):89. 10.1186/s12974-024-03085-9. 10.1186/s12974-024-03085-9 PMC11007930 38600510

[37] Reynolds JA, Torz L, Cummins L, Stock AD, Ben-Zvi A, Putterman C. Blood–CSF barrier clearance of ABC transporter substrates is suppressed by interleukin-6 in lupus choroid plexus spheroids. Fluids Barriers CNS. 2025;22(1):15. 10.1186/s12987-025-00628-x. 39934822 10.1186/s12987-025-00628-x PMC11816793

[38] Ransohoff RM, Brown MA. Innate immunity in the central nervous system. J Clin Invest. 2012;122(4):1164–71. 10.1172/jci58644. 22466658 10.1172/JCI58644 PMC3314450

[39] Block ML, Zecca L, Hong JS. Microglia-mediated neurotoxicity: uncovering the molecular mechanisms. Nat Rev Neurosci. 2007;8(1):57–69. 10.1038/nrn2038. 17180163 10.1038/nrn2038

[40] Zeis T, Enz L, Schaeren-Wiemers N. The immunomodulatory oligodendrocyte. Brain Res. 2016;1641(Pt A):139–48. 10.1016/j.brainres.2015.09.021. 26423932 10.1016/j.brainres.2015.09.021

[41] Lin SL, Carroll MC. Neuroimmune mechanisms of neuropsychiatric systemic lupus erythematosus. Curr Opin Immunol. 2025;96:102608. 10.1016/j.coi.2025.102608. 40683115 10.1016/j.coi.2025.102608 PMC12516810

[42] Chen GY, Nuñez G. Sterile inflammation: sensing and reacting to damage. Nat Rev Immunol. 2010;10(12):826–37. 10.1038/nri2873. 21088683 10.1038/nri2873 PMC3114424

[43] Kono H, Rock KL. How dying cells alert the immune system to danger. Nat Rev Immunol. 2008;8(4):279–89. 10.1038/nri2215. 18340345 10.1038/nri2215 PMC2763408

[44] Scaffidi P, Misteli T, Bianchi ME. Release of chromatin protein HMGB1 by necrotic cells triggers inflammation. Nature. 2002;418(6894):191–5. 10.1038/nature00858. 12110890 10.1038/nature00858

[45] Gao HM, Zhou H, Zhang F, Wilson BC, Kam W, Hong JS. HMGB1 acts on microglia Mac1 to mediate chronic neuroinflammation that drives progressive neurodegeneration. J Neurosci. 2011;31(3):1081–92. 10.1523/jneurosci.3732-10.2011. 21248133 10.1523/JNEUROSCI.3732-10.2011 PMC3046932

[46] Paudel YN, Shaikh MF, Chakraborti A, et al. HMGB1: a common biomarker and potential target for TBI, neuroinflammation, epilepsy, and cognitive dysfunction. Front Neurosci. 2018;12:628. 10.3389/fnins.2018.00628. 30271319 10.3389/fnins.2018.00628 PMC6142787

[47] Liu T, Son M, Diamond B. HMGB1 in systemic lupus erythematosus. Front Immunol. 2020;11:1057. 10.3389/fimmu.2020.01057. 32536928 10.3389/fimmu.2020.01057 PMC7267015

[48] Liddelow SA, Guttenplan KA, Clarke LE, et al. Neurotoxic reactive astrocytes are induced by activated microglia. Nature. 2017;541(7638):481–7. 10.1038/nature21029. 28099414 10.1038/nature21029 PMC5404890

[49] Perry VH, Nicoll JA, Holmes C. Microglia in neurodegenerative disease. Nat Rev Neurol. 2010;6(4):193–201. 10.1038/nrneurol.2010.17. 20234358 10.1038/nrneurol.2010.17

[50] Gao HM, Hong JS. Why neurodegenerative diseases are progressive: uncontrolled inflammation drives disease progression. Trends Immunol. 2008;29(8):357–65. 10.1016/j.it.2008.05.002. 18599350 10.1016/j.it.2008.05.002 PMC4794280

[51] Latz E, Xiao TS, Stutz A. Activation and regulation of the inflammasomes. Nat Rev Immunol. 2013;13(6):397–411. 10.1038/nri3452. 23702978 10.1038/nri3452 PMC3807999

[52] Ginhoux F, Greter M, Leboeuf M, et al. Fate mapping analysis reveals that adult microglia derive from primitive macrophages. Science. 2010;330(6005):841–5. 10.1126/science.1194637. 20966214 10.1126/science.1194637 PMC3719181

[53] Farina C, Aloisi F, Meinl E. Astrocytes are active players in cerebral innate immunity. Trends Immunol. 2007;28(3):138–45. 10.1016/j.it.2007.01.005. 17276138 10.1016/j.it.2007.01.005

[54] Shen W, Song S, Zhao X, Li D, Liu J, Yue S. Transcription factor RUNX2 participates in astrocyte-mediated neuropathic pain via transcriptional activation of Ccl2. Biochem Biophys Res Commun. 2025;777:152263. 10.1016/j.bbrc.2025.152263. 40609207 10.1016/j.bbrc.2025.152263

[55] Pekny M, Pekna M. Astrocyte reactivity and reactive astrogliosis: costs and benefits. Physiol Rev. 2014;94(4):1077–98. 10.1152/physrev.00041.2013. 25287860 10.1152/physrev.00041.2013

[56] Schafer DP, Lehrman EK, Kautzman AG, et al. Microglia sculpt postnatal neural circuits in an activity and complement-dependent manner. Neuron. 2012;74(4):691–705. 10.1016/j.neuron.2012.03.026. 22632727 10.1016/j.neuron.2012.03.026 PMC3528177

[57] Stevens B, Allen NJ, Vazquez LE, et al. The classical complement cascade mediates CNS synapse elimination. Cell. 2007;131(6):1164–78. 10.1016/j.cell.2007.10.036. 18083105 10.1016/j.cell.2007.10.036

[58] Hong S, Beja-Glasser VF, Nfonoyim BM, et al. Complement and microglia mediate early synapse loss in Alzheimer’s mouse models. Science. 2016;352(6286):712–6. 10.1126/science.aad8373. 27033548 10.1126/science.aad8373 PMC5094372

[59] Bialas AR, Presumey J, Das A, et al. Microglia-dependent synapse loss in type I interferon-mediated lupus. Nature. 2017;546(7659):539–43. 10.1038/nature22821. 28614301 10.1038/nature22821

[60] Luoma A, Matilainen M, Tuisku JM, et al. Synaptic density in multiple sclerosis: an in vivo study using [(11)C]UCB-J-PET imaging. Neurol Neuroimmunol Neuroinflamm. 2025;12(5):e200435. 10.1212/nxi.0000000000200435. 40609051 10.1212/NXI.0000000000200435 PMC12227148

[61] Miller AH, Raison CL. The role of inflammation in depression: from evolutionary imperative to modern treatment target. Nat Rev Immunol. 2016;16(1):22–34. 10.1038/nri.2015.5. 26711676 10.1038/nri.2015.5 PMC5542678

[62] Wichers MC, Koek GH, Robaeys G, Verkerk R, Scharpé S, Maes M. IDO and interferon-alpha-induced depressive symptoms: a shift in hypothesis from tryptophan depletion to neurotoxicity. Mol Psychiatry. 2005;10(6):538–44. 10.1038/sj.mp.4001600. 15494706 10.1038/sj.mp.4001600

[63] Guillemin GJ, Brew BJ, Noonan CE, Takikawa O, Cullen KM. Indoleamine 2,3 dioxygenase and quinolinic acid immunoreactivity in Alzheimer’s disease hippocampus. Neuropathol Appl Neurobiol. 2005;31(4):395–404. 10.1111/j.1365-2990.2005.00655.x. 16008823 10.1111/j.1365-2990.2005.00655.x

[64] Tavares RG, Tasca CI, Santos CE, Wajner M, Souza DO, Dutra-Filho CS. Quinolinic acid inhibits glutamate uptake into synaptic vesicles from rat brain. NeuroReport. 2000;11(2):249–53. 10.1097/00001756-200002070-00005. 10674464 10.1097/00001756-200002070-00005

[65] Raison CL, Dantzer R, Kelley KW, et al. CSF concentrations of brain tryptophan and kynurenines during immune stimulation with IFN-alpha: relationship to CNS immune responses and depression. Mol Psychiatry. 2010;15(4):393–403. 10.1038/mp.2009.116. 19918244 10.1038/mp.2009.116 PMC2844942

[66] Li Y, Eskelund AR, Zhou H, Budac DP, Sánchez C, Gulinello M. Behavioral deficits are accompanied by immunological and neurochemical changes in a mouse model for neuropsychiatric lupus (NP-SLE). Int J Mol Sci. 2015;16(7):15150–71. 10.3390/ijms160715150. 26151848 10.3390/ijms160715150 PMC4519892

[67] Hamby ME, Sofroniew MV. Reactive astrocytes as therapeutic targets for CNS disorders. Neurotherapeutics. 2010;7(4):494–506. 10.1016/j.nurt.2010.07.003. 20880511 10.1016/j.nurt.2010.07.003 PMC2952540

[68] Lipton SA. Paradigm shift in neuroprotection by NMDA receptor blockade: memantine and beyond. Nat Rev Drug Discov. 2006;5(2):160–70. 10.1038/nrd1958. 16424917 10.1038/nrd1958

[69] Arinuma Y, Yanagida T, Hirohata S. Association of cerebrospinal fluid anti-NR2 glutamate receptor antibodies with diffuse neuropsychiatric systemic lupus erythematosus. Arthritis Rheum. 2008;58(4):1130–5. 10.1002/art.23399. 18383393 10.1002/art.23399

[70] Diamond B, Huerta PT, Mina-Osorio P, Kowal C, Volpe BT. Losing your nerves? Maybe it’s the antibodies. Nat Rev Immunol. 2009;9(6):449–56. 10.1038/nri2529. 19424277 10.1038/nri2529 PMC2783680

[71] Monje ML, Toda H, Palmer TD. Inflammatory blockade restores adult hippocampal neurogenesis. Science. 2003;302(5651):1760–5. 10.1126/science.1088417. 14615545 10.1126/science.1088417

[72] Ekdahl CT, Claasen JH, Bonde S, Kokaia Z, Lindvall O. Inflammation is detrimental for neurogenesis in adult brain. Proc Natl Acad Sci U S A. 2003;100(23):13632–7. 10.1073/pnas.2234031100. 14581618 10.1073/pnas.2234031100 PMC263865

[73] Gulinello M, Putterman C. The MRL/lpr mouse strain as a model for neuropsychiatric systemic lupus erythematosus. J Biomed Biotechnol. 2011;2011:207504. 10.1155/2011/207504. 21331367 10.1155/2011/207504 PMC3038428

[74] Appenzeller S, Carnevalle AD, Li LM, Costallat LT, Cendes F. Hippocampal atrophy in systemic lupus erythematosus. Ann Rheum Dis. 2006;65(12):1585–9. 10.1136/ard.2005.049486. 16439436 10.1136/ard.2005.049486 PMC1798450

[75] Zhao L, Hu X, Xiao F, Zhang X, Zhao L, Wang M. Mitochondrial impairment and repair in the pathogenesis of systemic lupus erythematosus. Front Immunol. 2022;13:929520. 10.3389/fimmu.2022.929520. 35958572 10.3389/fimmu.2022.929520 PMC9358979

[76] Morand EF, Furie R, Tanaka Y, et al. Trial of anifrolumab in active systemic lupus erythematosus. N Engl J Med. 2020;382(3):211–21. 10.1056/NEJMoa1912196. 31851795 10.1056/NEJMoa1912196

[77] Wu YW, Tang W, Zuo JP. Toll-like receptors: potential targets for lupus treatment. Acta Pharmacol Sin. 2015;36(12):1395–407. 10.1038/aps.2015.91. 26592511 10.1038/aps.2015.91 PMC4816237

[78] Ringelstein M, Ayzenberg I, Lindenblatt G, et al. Interleukin-6 receptor blockade in treatment-refractory MOG-IgG-associated disease and neuromyelitis optica spectrum disorders. Neurol Neuroimmunol Neuroinflamm. 2022. 10.1212/nxi.0000000000001100. 34785575 10.1212/NXI.0000000000001100 PMC8596357

[79] Prakhova LN, Krasnov VS, Kasatkin DS, Korobko DS. Local experience of IL-6 pathway inhibition with satralizumab for patients with neuromyelitis optica spectrum disorder. Zh Nevrol Psikhiatr Im S S Korsakova. 2022. 10.17116/jnevro202212207268. 35912559 10.17116/jnevro202212207268

[80] Kübler L, Bittmann I, Kuipers JG. Macrophage activation syndrome triggered by active systemic lupus erythematosus: successful treatment by interleukin-1 inhibition (anakinra). Z Rheumatol. 2020;79(10):1040–5. 10.1007/s00393-020-00836-w. 32804304 10.1007/s00393-020-00836-w

[81] Petri M, Bruce IN, Dörner T, et al. Baricitinib for systemic lupus erythematosus: a double-blind, randomised, placebo-controlled, phase 3 trial (SLE-BRAVE-II). Lancet. 2023;401(10381):1011–9. 10.1016/s0140-6736(22)02546-6. 36848919 10.1016/S0140-6736(22)02546-6

[82] Yin J, Hou Y, Wang C, Qin C. Clinical outcomes of baricitinib in patients with systemic lupus erythematosus: pooled analysis of SLE-BRAVE-I and SLE-BRAVE-II trials. PLoS ONE. 2025;20(4):e0320179. 10.1371/journal.pone.0320179. 40305472 10.1371/journal.pone.0320179 PMC12043178

[83] Huo R, Huang X, Yang Y, Lin J. Potential use of Janus kinase inhibitors in the treatment of systemic lupus erythematosus. J Inflamm Res. 2023;16:1471–8. 10.2147/jir.S397639. 37051062 10.2147/JIR.S397639 PMC10084827

[84] Spangenberg EE, Lee RJ, Najafi AR, et al. Eliminating microglia in Alzheimer’s mice prevents neuronal loss without modulating amyloid-*β* pathology. Brain. 2016;139(Pt 4):1265–81. 10.1093/brain/aww016. 26921617 10.1093/brain/aww016 PMC5006229

[85] Olmos-Alonso A, Schetters ST, Sri S, et al. Pharmacological targeting of CSF1R inhibits microglial proliferation and prevents the progression of Alzheimer’s-like pathology. Brain. 2016;139(Pt 3):891–907. 10.1093/brain/awv379. 26747862 10.1093/brain/awv379 PMC4766375

[86] Ponce J, de la Ossa NP, Hurtado O, et al. Simvastatin reduces the association of NMDA receptors to lipid rafts: a cholesterol-mediated effect in neuroprotection. Stroke. 2008;39(4):1269–75. 10.1161/strokeaha.107.498923. 18323503 10.1161/STROKEAHA.107.498923

[87] Cheng G, Wei L, Zhi-Dan S, Shi-Guang Z, Xiang-Zhen L. Atorvastatin ameliorates cerebral vasospasm and early brain injury after subarachnoid hemorrhage and inhibits caspase-dependent apoptosis pathway. BMC Neurosci. 2009;10:7. 10.1186/1471-2202-10-7. 19159448 10.1186/1471-2202-10-7 PMC2651177

[88] Kluger MS, Clark PR, Tellides G, Gerke V, Pober JS. Claudin-5 controls intercellular barriers of human dermal microvascular but not human umbilical vein endothelial cells. Arterioscler Thromb Vasc Biol. 2013;33(3):489–500. 10.1161/atvbaha.112.300893. 23288152 10.1161/ATVBAHA.112.300893 PMC3655806

[89] Burek M, Arias-Loza PA, Roewer N, Förster CY. Claudin-5 as a novel estrogen target in vascular endothelium. Arterioscler Thromb Vasc Biol. 2010;30(2):298–304. 10.1161/atvbaha.109.197582. 19910637 10.1161/ATVBAHA.109.197582

[90] Hoyles L, Pontifex MG, Rodriguez-Ramiro I, et al. Regulation of blood-brain barrier integrity by microbiome-associated methylamines and cognition by trimethylamine N-oxide. Microbiome. 2021;9(1):235. 10.1186/s40168-021-01181-z. 34836554 10.1186/s40168-021-01181-z PMC8626999

[91] Gaire BP, Sapkota A, Song MR, Choi JW. Lysophosphatidic acid receptor 1 (LPA(1)) plays critical roles in microglial activation and brain damage after transient focal cerebral ischemia. J Neuroinflamm. 2019;16(1):170. 10.1186/s12974-019-1555-8. 10.1186/s12974-019-1555-8 PMC6701099 31429777

[92] Kariuki SN, Franek BS, Kumar AA, et al. Trait-stratified genome-wide association study identifies novel and diverse genetic associations with serologic and cytokine phenotypes in systemic lupus erythematosus. Arthritis Res Ther. 2010;12(4):R151. 10.1186/ar3101. 20659327 10.1186/ar3101 PMC2945049

[93] Nagata W, Takayama E, Nakagawa K, et al. Treatment with lysophosphatidic acid improves glomerulonephritis through the suppression of macrophage activation in a murine model of systemic lupus erythematosus. Clin Exp Rheumatol. 2024. 10.55563/clinexprheumatol/ov6027. 38436267 10.55563/clinexprheumatol/ov6027

[94] Nagata W, Koizumi A, Nakagawa K, et al. Treatment with lysophosphatidic acid prevents microglial activation and depression-like behaviours in a murine model of neuropsychiatric systemic lupus erythematosus. Clin Exp Immunol. 2023;212(2):81–92. 10.1093/cei/uxad010. 36718978 10.1093/cei/uxad010 PMC10128163

[95] Li H, Wang D, Zhang H, et al. Lysophosphatidic acid stimulates cell migration, invasion, and colony formation as well as tumorigenesis/metastasis of mouse ovarian cancer in immunocompetent mice. Mol Cancer Ther. 2009;8(6):1692–701. 10.1158/1535-7163.Mct-08-1106. 19509252 10.1158/1535-7163.MCT-08-1106

[96] Benesch MG, Ko YM, McMullen TP, Brindley DN. Autotaxin in the crosshairs: taking aim at cancer and other inflammatory conditions. FEBS Lett. 2014;588(16):2712–27. 10.1016/j.febslet.2014.02.009. 24560789 10.1016/j.febslet.2014.02.009

[97] Nagata W, Gotoh M, Koizumi A, et al. Two-carba cyclic phosphatidic acid treatment promotes phenotypic switch from M1 to M2 microglia and prevents behavioral abnormalities in a mouse model of neuropsychiatric systemic lupus erythematosus. Hum Cell. 2023;36(6):2006–15. 10.1007/s13577-023-00964-w. 37540445 10.1007/s13577-023-00964-w

[98] Wang X, Tang J, Lu F, et al. A novel nomogram based on the identification of sTREM2 as a biomarker to predict developing neuropsychiatric systemic lupus erythematosus in lupus patients. Clin Exp Rheumatol. 2025. 10.55563/clinexprheumatol/1tcgmj. 40371560 10.55563/clinexprheumatol/1tcgmj

